# Invasive Basidiobolomycosis Presenting as Retroperitoneal Fibrosis: A Case Report

**DOI:** 10.3390/ijerph17020535

**Published:** 2020-01-15

**Authors:** Abdulmalek Alsharidah, Yahya Mahli, Nayef Alshabyli, Mohammed Alsuhaibani

**Affiliations:** 1 Department of Radiology, King Fahad Medical City, Riyadh 11525, Saudi Arabia; mahalli777@hotmail.com; 2 Department of Radiology, Prince Sultan Military Medical City, Riyadh 11159, Saudi Arabia; nayef663@gmail.com; 3 Department of Pediatrics, College of Medicine, Qassim University, Qassim 51452, Saudi Arabia; msuhaibani@qumed.edu.sa

**Keywords:** basidiobolomycosis, retroperitoneal, fungal infection, mycoses

## Abstract

Basidiobolomycosis is an uncommon emerging fungal infection caused by *Basidiobolus ranarum*. It frequently causes cutaneous infection, but it rarely infects visceral tissues in humans. Here, a 39-year-old previously healthy woman presented with severe left-sided abdominal pain and weight loss. She had visited several hospitals and had provisionally been diagnosed as having either a retroperitoneal malignancy or retroperitoneal fibrosis before being referred to our hospital. Abdominal computerized tomography and biopsy of the retroperitoneal mass revealed retroperitoneal basidiobolomycosis infection. She was started on antifungal treatment. This led to significant improvement, without surgical intervention. Gastrointestinal basidiobolomycosis can present in many forms, commonly involving the colon and liver with multifocal inflammatory masses. Nonetheless, retroperitoneal basidiobolomycosis presentation is extremely rare and should be considered in the differential diagnosis of a retroperitoneal mass with eosinophilia.

## 1. Introduction

Basidiobolomycosis is a rare emerging fungal infection, which was first described in humans in 1956, and is caused by *Basidiobolus ranarum* [1,2]. This organism is a commensal in certain amphibians such as frogs and toads. Additionally, it is found in fish, reptiles, and bats [3]. The mode of transmission to humans is not fully understood. The subcutaneous form usually occurs in tropical and subtropical regions, and possible routes of infection include skin penetration after a skin injury, scratches, insect bites, the practice of using decaying leaves as toilet tissue after defection, especially in young children [4,5]. Ingestion of soil, animal feces, or food contaminated with *Basidiobolus ranarum* are possible routes of transmission of gastrointestinal basidiobolomycosis (GIB) [6].

GIB is uncommon and affects young and immunocompetent individuals, particularly children [6]. It typically involves the colon, small bowel, liver, or stomach, but not the retroperitoneum. The majority of cases that have been reported have been from Saudi Arabia, United States, Iran, and Brazil [7,8].

Diagnosis of GIB is difficult to make in the initial presentation due to the non-specific nature of the abdominal symptoms and radiological findings, which usually mimic malignancy, inflammatory bowel disease, or diverticulitis. The key laboratory finding is eosinophilia and the histopathological appearance of fungal hyphae surrounded by eosinophilic infiltration (Splendore–Hoeppli phenomenon) in an individual from an endemic areas [9,10]. Herein we report a case of a young Saudi woman with retroperitoneal basidiobolomycosis who was treated successfully with an antifungal agent, without surgical intervention.

## 2. Case Report

A previously healthy 39-year-old woman from the southern part of Saudi Arabia, who had no medical conditions of note other than hypertension, for which she was being treated with amlodipine, presented to our hospital with progressive, severe, left-abdominal pain and a history of weight loss over the previous two months without history of fever. She had had a laparoscopic removal of an ovarian cyst two years prior to her presentation. She had no history of trauma or of any unusual habits. She had sought care from several other hospitals before being referred to our hospital for further management as a case of retroperitoneal fibrosis. The physical examination showed no abnormalities and no palpable masses.

We initially worked-up the patient as a case of retroperitoneal fibrosis. The initial laboratory results revealed white blood cells (WBC) 5.47 × 10^9^ cells/L, eosinophils 15.7%, absolute eosinophils 0.86 × 10^9^ cells/L, erythrocyte sedimentation rate (ESR) 101 mm/h, and C-reactive protein (CRP) 8.63 mg/L. Additionally, immunological testing revealed that her antinuclear antibody (ANA) was negative, and her immunoglobulin G (IgG) subclasses were elevated as follows: IgG1 12.5, IgG2 9.49, IgG3 1.76, and IgG4 6.7 g/L.

**Figure 1:**
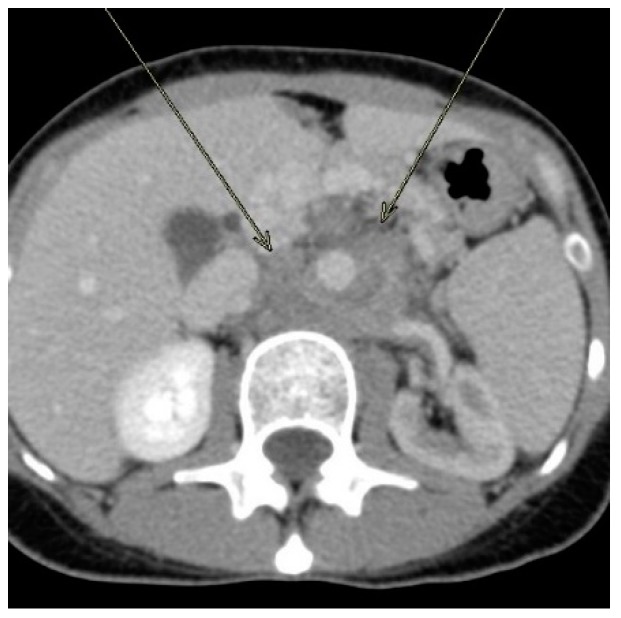
Axial computerized tomography of the abdomen with intravenous contrast showing a large upper retroperitoneal mass surrounding the aorta. It encased the aorta, celiac artery, superior mesenteric artery, and left renal artery, causing severe narrowing/occlusion of these vessels. It also encased the left renal vein, which was completely occluded and replaced by collaterals. The mass extended to the root of the mesentery around the superior and inferior mesenteric arteries. The left kidney showed reduced enhancement due to involvement of the left renal vessels. The tumor invaded the left adrenal gland, but the colon was not involved.

A computerized tomography (CT) scan of her abdomen (Figure 1) revealed a large, retroperitoneal, hypodense, ill-defined mass surrounding the aorta and encasing the celiac artery, superior mesenteric artery, and left renal artery, causing severe narrowing of these vessels. It was also invading and occluding her left renal vein.

**Figure 2:**
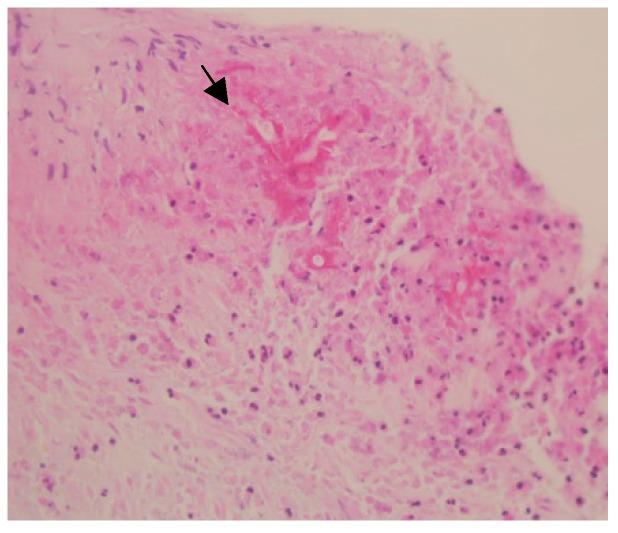
Histopathological appearance of *Basidiobolus ranarum*, showing broad, branching fungal hyphae (black arrow).

We performed CT-guided biopsy of the mass. The histopathological evaluation revealed broad and branching fungal hyphae with presence of eosinophilic sheets, an appearance characteristic of basidiobolomycosis (Figure 2). We initiated antifungal treatment (oral itraconazole 200 mg twice daily) without any surgical intervention. A follow-up CT scan, performed 5 months later showed an excellent response to treatment, with a >60% reduction in the size of the retroperitoneal mass. In addition, laboratory investigation showed the following improvements: eosinophils 7.3% (compared to 15.7%), absolute eosinophils 0.68 × 10^9^ cells/L (compared to 0.86 × 10^9^ cells/L), ESR 42 mm/h (compared to 101 mm/h), and CRP 0.45 mg/L (compared to 8.63 mg/L). Currently, she is still on treatment and undergoing follow-up.

## 3. Discussion

GIB is an emerging fungal infection of visceral and gastrointestinal tissues. The first case of GIB was reported in a Nigerian child in 1964 [11]. Most of the cases reported have been from Saudi Arabia, and have occurred in a young age group [12]. Making the initial diagnosis is usually difficult and requires a high index of suspicion. Malignancy, inflammatory bowel disease, diverticulitis, and tuberculosis are the most common differential diagnoses [9,10,13]. In small case-control study, antacids such as ranitidine, and smoking were identified as a potential risk factors for GIB [14].

The diagnosis is difficult because of its non-specific clinical presentation, which is usually an insidious onset abdominal pain, fever, weight loss, diarrhea, or the presence of an abdominal mass. The most commonly affected abdominal organs are colon (84%), small bowel (32%), liver (21%), and rarely, other organs such as the stomach may be involved [7,12,15]. Moreover, concurrent subcutaneous and visceral basidiobolomycosis has been described in renal-transplanted adult patients [16].

Peripheral blood eosinophilia was present in most reported cases. Culture is required for the definitive diagnosis, the majority of cases are diagnosed based on the characteristic histopathological appearance of the infected tissue, which shows fungal hyphae surrounded by eosinophilia (Splendore–Hoeppli phenomenon) [17]. Recently, PCR has been shown to be a highly sensitive and specific test, but it is not widely used due to the scarcity of the disease [18]. In this case, the patient had high eosinophilia and the biopsy specimen of the lesion had a characteristic histopathological appearance. Culture was not performed because we initially suspected that the patient had retroperitoneal fibrosis.

The radiological manifestations of basidiobolomycosis vary according to the organ affected. CT is the most common radiological modality used for diagnosis. The most common abdominal finding is colonic masses. Masses in the liver and loops of small bowel loops are also commonly seen. Less frequent manifestations include involvement of the kidneys, gallbladder, pancreas, and retroperitoneum [7]. Additionally, life-threatening consequences such as gastrointestinal tract perforation, abdominal collections, and hydronephrosis have been reported [19,20]. In this case, only the retroperitoneum was involved, therefore the retroperitoneal fibrosis was initially specified as the probable diagnosis, giving its appearance on CT. In this case, the left kidney was affected indirectly by the compromised blood supply due to disease involvement of the left renal vessels.

The prevalence of GIB in the southwestern region of Saudi Arabia (Tohama) is increasing, primarily due to an increased awareness of the disease. Therefore, the diagnosis should be considered in any patient from this endemic area who present with an inflammatory abdominal mass. GIB has a case fatality rate of up to 20% according to an analysis of retrospective data [10]. Hence, a high index of suspicion, early diagnosis, and antifungal treatment are crucial.

The treatment of choice for abdominal basidiobolomycosis is still a matter of debate. A combination of surgical resection of the affected tissue followed by long-term antifungal medication has been the preferred treatment previously [21]. However, there has been a recent shift toward treating the condition with antifungal medication alone, particularly with antifungals from the azole group. Medical treatment for 6–12 months, without surgical intervention, has shown to produce good results in both children and adults [22,23,24]. Therefore, surgery can be avoided, depending on the patient’s critical status and response to medication.

## 4. Conclusions

GIB can present in many forms, commonly involving the colon and liver with multifocal inflammatory masses. Nonetheless, retroperitoneal basidiobolomycosis presentation is extremely rare and should be considered in the differential diagnosis of a retroperitoneal mass with eosinophilia.
